# Characterization of Biosurfactant Produced during Degradation of Hydrocarbons Using Crude Oil As Sole Source of Carbon

**DOI:** 10.3389/fmicb.2017.00279

**Published:** 2017-02-22

**Authors:** Kaustuvmani Patowary, Rupshikha Patowary, Mohan C. Kalita, Suresh Deka

**Affiliations:** ^1^Environmental Biotechnology Laboratory, Life Sciences Division, Institute of Advanced Study in Science and TechnologyGuwahati, India; ^2^Department of Biotechnology, Gauhati UniversityGuwahati, India

**Keywords:** biosurfactant, crude oil, PAHs, biodegradation, rhamnolipid, *Pseudomonas aeruginosa* PG1

## Abstract

Production and spillage of petroleum hydrocarbons which is the most versatile energy resource causes disastrous environmental pollution. Elevated oil degrading performance from microorganisms is demanded for successful microbial remediation of those toxic pollutants. The employment of biosurfactant-producing and hydrocarbon-utilizing microbes enhances the effectiveness of bioremediation as biosurfactant plays a key role by making hydrocarbons bio-available for degradation. The present study aimed the isolation of a potent biosurfactant producing indigenous bacteria which can be employed for crude oil remediation, along with the characterization of the biosurfactant produced during crude oil biodegradation. A potent bacterial strain *Pseudomonas aeruginosa* PG1 (identified by 16s rDNA sequencing) was isolated from hydrocarbon contaminated soil that could efficiently produce biosurfactant by utilizing crude oil components as the carbon source, thereby leading to the enhanced degradation of the petroleum hydrocarbons. Strain PG1 could degrade 81.8% of total petroleum hydrocarbons (TPH) after 5 weeks of culture when grown in mineral salt media (MSM) supplemented with 2% (v/v) crude oil as the sole carbon source. GCMS analysis of the treated crude oil samples revealed that *P. aeruginosa* PG1 could potentially degrade various hydrocarbon contents including various PAHs present in the crude oil. Biosurfactant produced by strain PG1 in the course of crude oil degradation, promotes the reduction of surface tension (ST) of the culture medium from 51.8 to 29.6 mN m^−1^, with the critical micelle concentration (CMC) of 56 mg L^−1^. FTIR, LC-MS, and SEM-EDS studies revealed that the biosurfactant is a rhamnolipid comprising of both mono and di rhamnolipid congeners. The biosurfactant did not exhibit any cytotoxic effect to mouse L292 fibroblastic cell line, however, strong antibiotic activity against some pathogenic bacteria and fungus was observed.

## Introduction

Crude petroleum oil and its derivatives are considered as one of the most pervasive environmental pollutants because they produce a problem of increasing enormity around the globe (Okoh and Trejo-Hernandez, 2006). The profuseness of petroleum in any petroleum producing locality arises both as a blessing and a curse, because unfortunately most of the crude oil drilling sites and storage facilities are based at the periphery of human settlement. In the process of oil exploration, collection and transportation from the drilling site, leakage of crude oils results in wide-ranging contamination of adjacent agricultural fields and water bodies. Accidental and deliberate spillage and instinctive environmental contamination have been a major threat to the ecosystem and biota through the transfer of toxic organic materials including complex mixture of aliphatics, aromatics (including polycyclic aromatic hydrocarbons, i.e., PAHs), nitrogen, sulfur, metals etc. into the food chain (Reddy et al., 2011; Wang et al., 2015). Amongst them, PAHs are considered as critical environmental pollutants due to their extreme resistance to various methods of bioconversion because of their characteristic chemical stability (Hwang et al., 2007). The various components of crude petroleum oil can trigger multiple toxic effects including sub-lethal chronic toxicity, acute lethal toxicity or both, as determined by the exposure type and the organism exposed (Orisakwe et al., 2004; Hwang et al., 2007). Spillage of oil can often lead to both immediate and long-term environmental damage (Martínez-Palou et al., 2013). Furthermore, this problem is more aggravated because of unsafe disposal methods owing to the associated higher cost of safe and proper disposal (Rahman et al., 2003). Thus, these detrimental hydrocarbon pollutants make the development of a remediation technology essential for cleaning up polluted sites. As compared to other strategies adopted to treat crude petroleum contamination, microbial remediation is recognized as one of the effective, eco-friendly and inexpensive technologies (Bento et al., 2005). Free-living and ubiquitous microorganism, bacteria have long been considered as one of the predominant hydrocarbon degrading agents (Chi et al., 2012; Dasgupta et al., 2013). Although there exist numerous hydrocarbon-degraders in nature, the growth of most of them is hindered by a number of factors like recalcitrant nature of substrate and limited availability of organic compounds in aqueous systems which ultimately constrains their utilization by the existing micro-flora (Calvo et al., 2009). A suitable method that can be adopted to speed up the bioremediation of sites contaminated with hydrocarbon, is the involvement of biosurfactant producing hydrocarbon degrader microorganism.

A plethora of microorganism have been reported as producers of biosurfactants which are of diverse chemical compositions such as glycolipids, fatty acids, lipopeptides and lipoproteins, phospholipids, and neutral lipids (Cameotra and Makkar, 2010). Glycolipids are biosurfactants with different structural variations having wide range of applicability. These stable but readily biodegradable biosurfactants are amphiphilic in nature in which alkyl chains are linked to sugar molecules giving those hydrophilic and hydrophobic regions (Costa et al., 2010). Biosurfactant reduces surface tension (ST) or interfacial tension of an interface, depending whether it is a water/air or water/oil interface. In water/oil interface, biosurfactant molecule generates a new surface area by forming a surfactant oriented monolayer around the hydrocarbon particle with hydrophobic tail of the surfactant pointing out to the liquid phase (Harkins and Jordan, 1930). This leads to increase in surface area of hydrocarbon substrate and facilitates emulsification. The entire phenomena enhances the bioavailability of contaminants for microbial degradation through better solubilization of hydrocarbons in water or water in hydrocarbons (Banat et al., 2014). Due to the lower toxicity and biodegradable nature in comparison to their synthetic counterparts, biosurfactants are considered to be more suitable for environmental applications such as hydrocarbon remediation (Oberbremer et al., 1990). In the hydrocarbon degradation process, some microorganisms secrete biosurfactants into the growth medium and alter cell surface property by reducing the cell surface hydrophobicity (Ramos-Gonzalez et al., 1991). The poor bioavailability of hydrocarbon components is considered as a major rate limiting factor in the hydrocarbon remediation process (Das et al., 2014). The biosurfactant molecules enhance the solubility of these sparsely soluble hydrophobic pollutants through emulsification, thereby leading their better bioavailability for the existing micro-flora (Das et al., 2014). Hydrocarbon degraders are in fact well-known for their potential to produce biosurfactants *in situ* which promote their survival in hydrophobic compound dominated environments (Ganesh and Lin, 2009). The enhancement of petroleum oil degradation by dint of biosurfactant production ability has been well-studied in members of several bacterial genera like *Pseudomonas, Bacillus, Acenetobacter, Alcaligenes, Rhodococcus, Corynebacterium* etc (Cameotra and Makkar, 2010; Abbasian et al., 2016). The application of microbes posing capacity to degrade hydrocarbons along with the production of biosurfactants can effectively expedite the bioremediation of hydrocarbon polluted environment (Kumar et al., 2006). Although, the chemical and physical properties of some biosurfactant classes are well-studied, it's very important to characterize the biosurfactants produced during the hydrocarbon degradation process as such type of study is very sparse (Chandankere et al., 2014). Therefore, the main intend of this work is to evaluate whether the screened hydrocarbon degrader bacteria could produce biosurfactant during the degradation process by utilizing the crude oil components and to characterize the biosurfactant that might be produced. Such a study will aid us in understanding the role of biosurfactant in hydrocarbon degradation process and provide a new dimension in the field of biosurfactant mediated bioremediation of hydrocarbon pollutants.

## Materials and methods

### Crude oil, soil samples, and chemicals

The model hydrocarbon contaminant, crude oil was obtained from Digboi Refinery, Assam, India and has been used throughout the study. Soil Samples for bacterial isolation were collected from an oil logging area of “Bajali automobiles,” a garage situated in Pathsala (26.4994°N, 91.1793°E), Assam, India. Media and chemicals of purity grade from Himedia, Merck and Sigma have been used throughout the study.

### Microorganism

For bacterial isolation, 1 gm of hydrocarbon contaminated soil samples was inoculated in mineral salt medium (MSM) containing 2% (v/v) crude oil as a carbon source for enrichment. The composition of the MSM used was the same as reported previously (Patowary et al., 2014). The pH of the medium was adjusted to 7.0 ± 0.2. The conical flasks were then incubated at 35°C at 150 rpm for 7 days. After 7 days, 1 ml inoculum was added to 100 ml of fresh MSM and incubated again under similar conditions for another 7 days to decrease unwanted microbial load. After this, 1 ml of the culture media was used for serial dilution followed by spreading of 100 μl from 10^−4^-10^−6^ diluted samples on nutrient agar plates and incubation of the plates at 35°C for 24 h. Bacterial colonies of different morphology were then selected and separately streaked on nutrient agar plates so as to obtain pure culture of the bacterial isolates. The isolates were maintained in nutrient agar slant and preserved in 30% glycerol storing at −80°C incubator.

### Screening of biosurfactant producing bacteria

Seed inoculums of same optical density (OD_600_ = 1.0) were prepared from all the bacterial isolates in nutrient broth (NB). An amount of 5 mL mother inoculums were inoculated into 500 mL Erlenmeyer flasks containing 100 mL MSM enriched with 2% (w/v) glucose as the carbon source and incubated at 35°C with shaking at 200 rpm. Production of biosurfactant of the bacterial isolates was assayed in terms of drop collapse assay and surface tension reduction of the culture medium.

### Drop collapse assay and surface tension measurement

Drop collapse assay was performed using crude oil as hydrocarbon substrate using a method described by Bodour and Miller-Maier with slight modification (Bodour and Miller-Maier, 1998). As the main intent of this study is degradation of crude oil, the same crude oil was taken as the substratefor this assay. A single drop of crude oil was set on a glass slide, following which a single drop of 48-h-grown culture broth was dropped onto the crude oil drop and drop collapse activity was observed.

ST reduction was measured after every 24 h up to 5th day of culture with a tensiometer (K11, Kruss, Germany). The isolates that could reduce ST of the culture medium below 35 mN m^−1^ were screened as efficient biosurfactant producers.

### Selection of efficient hydrocarbon degrading bacterial isolates

Among all the biosurfactant producing isolate, the most efficient crude oil degrader strain was selected depending on their growth in crude oil enriched condition. The screening was done as per the method mentioned by Rahman et al. with slight modification (Rahman et al., 2002). At first, seed inoculums of the bacterial isolates were prepared as mentioned earlier. For the screening, 5 mL seed culture of each bacterium was aseptically inoculated into 100 mL of sterilized mineral medium enriched with 2% (v/v) crude oil prepared in 500 mL Erlenmeyer flasks and kept in a shaking incubator for 7 days at 35°C and 200 rpm. A set of flasks containing the same composition of culture media was also maintained in same conditions as abiotic control where no inoculums were added. The bacterial growth in the medium of each flask at 0 and 7th day was estimated by taking optical density at 600 nm by UV-Vis spectrophotometer (Shimadzu UV-1800, Japan). The bacterial isolate showing maximum growth in crude oil containing media was selected for further studies.

### Identification of the bacterial strain

The most efficient isolate (designated as PG1) was identified according to standard biochemical tests (morphology and biochemistry) following Bergey's Manual of Systematic Bacteriology. Molecular identification in which the genomic DNA of the bacteria was extracted using standard protocol. The16S rDNA was PCR amplified using universal primer pair, 968F (AACGCGAAGAACCTTAC) and 1541R (AAGGAGGTGATCCAGCCGCA) (White et al., 1990). Polymerase chain reaction (PCR) was performed in a 25 μl volume in thermal cycler (Mastercycler Nexus gradient, Eppendorf, Germany) with a final concentration of 1X standard buffer, 1.5 m mol l^−1^ MgCl_2_, 0.2 μ mol l^−1^ each primer, 0.2 m mol l^−1^dNTPs and 0.25 U Taq DNA polymerase (Sigma Aldrich, USA) and 25 ng of template DNA. The PCR reaction conditions consisted of initial denaturation at 94°C for 5 min followed by 35 cycles of denaturation at 94°C for 30 s, annealing at 60°C for 30 s, extension at 72°C for 45 s, and a final extension at 72°C for 7 min. PCR products were analyzed on 1.2% agarose gel and visualized under Bio Doc-It Imaging System (UVP, USA). PCR products were purified with GenElute™ PCR Clean-Up Kit (Sigma Aldrich, USA). PCR products were sequenced bi-directionally using an automated sequencer by Beckman coulter (Genome Lab GeXP, Genetic Analysis System, and USA). 16S rDNA consensus sequence was used for Basic Local Alignment Search Tool (BLAST) analysis against the database in the National Centre for Biotechnology Information (NCBI) GenBank (www.ncbi.nlm.nih.gov). Sequence data were aligned using ClustalW and phylogenetic relationship among the strains were determined by the neighbor-joining method using MEGA 6 software (Thompson et al., 1994; Tamura et al., 2013).

### Degradation of crude oil by the selected bacterium

The selected bacterial strain, *Pseudomonas aeruginosa*PG1 was employed for degradation of crude oil in shake flask condition. Seed inoculums of the bacterial strain were prepared as mentioned earlier. Seed inoculums (5%) were aseptically added to respective flasks containing 100 ml MSM enriched with crude oil [2% (v/v)] as sole carbon source, followed by incubation at 35°C with and 200 rpm continuously till the 6th week. The growth of the strain in crude oil containing media was determined by estimating optical density of the media (OD_600nm_) before extraction of residual crude oil after every week of culture. Residual crude oil from the respective culture flask after every week of incubation was extracted using solvent extraction method by dichloromethane (DCM) and after solvent evaporation stored at previously weighed clean glass beaker. The remaining crude oil was quantified gravimetrically and thus crude oil degradation after every week of incubation was enumerated. The degradation percentage of hydrocarbon was calculated following the formula proposed by Ganesh and Lin (2009).

$$\begin{array}{l} \text{Hydrocarbon degradation}\left(\%\right)\\ =\left\{\frac{\begin{array}{c} \begin{array}{l} (\text{Weight of residual crude oil in}\\ \text{the abiotic control}) \end{array}\\ \begin{array}{l} -\;(\text{Weight of residual crude oil}\\ \text{in the test sample}) \end{array} \end{array}}{\begin{array}{l} \text{Original weight of crude oil}\\ \text{introduced} \end{array}}\right\}\times 100 \end{array}$$

The degradation of various hydrocarbon fractions was analyzed in the extracted crude oil samples obtained from the flasks containing MSM with 2% (v/v) crude oil which was inoculated with strain PG1 after 5 weeks of culture, where highest TPH degradation was observed in the gravimetric assay. For analysis and comparison, crude oil samples from the abiotic control were also extracted. Extracted crude oil samples were analyzed by GCMS to confirm the degradation efficacy of the strain by following the procedure given by Patowary et al. (2016b). The DCM extracted samples of treated crude oil and abiotic control were analyzed through a triple quadruple Gas Chromatograph-Mass Spectrometer (GC/MS TQ8030, Shimadzu, Japan) equipped with an auto-injector (AOC 20I, GC2010, E). For the detection of various petroleum hydrocarbons, the GC program was optimized and all analyses were carried out with the split ratio of 20:1. Helium was used as the carrier gas with a flow rate of 1.0 mL min^−1^, maintaining an injection temperature of 300°C. The column oven temperature was set at 60°C with a hold time of 5 min and was subsequently increased to 280°C with a ramp of 8°C min^−1^ with the final hold of 37 min. The mass spectrometric data were acquired in electron ionization mode (70 eV). The ion source temperature and interface temperature for MS were set at 230 and 310°C respectively. The mass range (m/z) was selected as 45–600 for the entire analysis. The chromatograms were analyzed with GC-MS solution software (version 4) and the compounds identification was performed using the NIST 11 library database.

### Extraction of biosurfactant

Biosurfactant produced in the course of crude oil degradation was extracted from the flasks after 5 weeks of culture, where highest TPH degradation was observed in the gravimetric assay. For the extraction of biosurfactant, cell-free supernatant was obtained through centrifugation of culture broth for 20 min at 10,000 rpm at 4°C which served as the source of crude biosurfactant. To amend the pH at 2, 6N HCl was added to the clear supernatant. The supernatant was acidified to pH 2 using 6N HCl and then stored at 4°C overnight. Biosurfactant was extracted from the refrigerated supernatant with ethyl acetate at room temperature continuously. A 1:1 mixer of ethyl acetate and supernatant was agitated vigorously and left stationary for phase separation. The organic phase was collected and then transferred to a rotary evaporator and a dark honey-colored viscous product was recovered after solvent evaporation at 40°C under reduced pressure (George and Jayachandran, 2008). The crude biosurfactant was quantified gravimetrically.

### Purification of biosurfactant and CMC determination

The purification of the crude biosurfactant was performed in a 26 × 3.3 cm^2^ chromatographic column containing 50 g of activated silica gel 60–120 (Merck, India) mesh chloroform (CHCl_3_) slurry. A 1 g crude biosurfactant sample was prepared in 5 ml of CHCl_3_ and loaded onto the column which was washed with chloroform until the neutral lipids were completely eluted. Mobile phase of CHCl_3_/CH_3_OH were applied in different ratio in sequence: 50:3 v/v (250 ml), 50:5 v/v (200 ml), and 50:50 v/v (100 ml) maintaining a flow rate of 1 ml min^−1^. Fractions of 20 ml were collected separately and biosurfactant detection in each fraction was done by measuring surface tension. Finally, the column was washed with 50:50 chloroform/methanol to remove remaining biosurfactant. All the biosurfactant containing fractions were mixed and dried under vacuum using a rotary evaporator to get the pure product.

The concentration of an amphiphilic component at which the formation of micelles is initiated in the solution corresponds to CMC (Abouseud et al., 2008). A concentration gradient ranging from 1.0 to 200 mg L^−1^ was prepared by dissolving extracted biosurfactant obtained from *P. aeruginosa* PG1 in distilled water for CMC calculation. By plotting the surface tension as a function of the biosurfactant concentration, the CMC was determined (Bonilla et al., 2005).

### Characterization of biosurfactant

Primary characterization of the biosurfactant was carried out using ninhydrin test, anthrone test, saponification test, and rhamnose test following the standardized methodology (Patowary et al., 2014, 2016a). Further characterization was achieved by using FTIR, LC-MS, and SEM-EDS analyses.

For chemical characterization of the biosurfactant FTIR analysis was performed. The column-purified biosurfactant was analyzed in NICOLET 6700 FTIR-Spectrophotometer (USA), in ATR (Attenuated total reflectance) mode considering a range of 500 to 4000 cm^−1^ for detection of functional groups and the bond type present.

Biosurfactant mixtures present in the purified product were separated and identification of various congeners (structural analog) were done by using LC-MS (Agilent Technologies 1260 Infinity LC and 6410 Triple Quad MS, USA). The column purified biosurfactant sample was dissolved in methanol and 2 μl aliquot was injected into ZORBAX C18 column (2.1 × 50 mm^2^). Flow rate of the LC was maintained at 0.20 mL min^−1^. Mobile phase of acetonitrile/water gradient (10–90%) with 0.01% formic acid was used in the column. ESI-MS was operated in positive ion mode and Agilent software was used for analysis. Through scanning the range of m/z 150–2000, full scan data were obtained where fragmentor voltage used was 135.0 V.

A thin layer of the column purified biosurfactant was prepared on a glass cover-slip followed by air drying. A coating of gold–palladium powder was done on the sample which was mounted on a stub over adhesive tape, using a Sputter Coater (SC-7625, EMITECH, India). Over the microscope support, the stub was positioned. By using a FE-SEM (Zeiss, P-Sigma, Germany) Scanning Electron Microscope the images were taken at 5 kV. By employing an X-ray detector, the energy dispersive X-ray spectroscopy (EDS) measurements were performed and analysis was done with INCA 4.15 EDS software (Oxford Instruments, UK).

### Emulsification test

The emulsification activity of the biosurfactant produced by PG1 on crude oil containing medium was evaluated by the method given by Cooper and Goldenberg (1987) against five hydrocarbon substrates, namely, n-hexadecane, kerosene, diesel oil, engine oil, and crude oil. Three milliliters of biosurfactant containing culture supernatant and 3 mL of respective oils were added in test tubes followed by rapid vigorous vortexing for 2 min. By adopting the formula given below the E24% was calculated.

$$\begin{array}{l} \text{E}24\%=(\text{Height of the emulsified layer}/\\ \text{ total height of liquid column})\times 100 \end{array}$$

### Cytotoxicity study of the biosurfactant

The toxicity level of the biosurfactant was determined with the help of a 3-(4, 5-dimethylthiazole-2-yl)-2, 5-diphenyltetrazolium bromide (MTT) dye conversion assay that was performed against mouse L292 fibroblastic cell line (collected from NCCS, Pune) (Kalita et al., 2015). The MTT assay detects cytotoxicity and proliferation by colorimetric method, which is based on the metabolic activity of viable cells in reducing tetrazolium salts (MTT) (Pathak et al., 2015). The L292 cells were cultured in 100 μL volume of Dulbecco's Modified Eagle Medium (DMEM) that was supplemented with 10% fetal bovine serum, on a 96-well cell culture plate by maintaining a density of 1 × 10^4^ cells. A series of different doses (50, 100, 200, and 250 μg) of column purified biosurfactant prepared in 100 μL DMEM without the serum were used to treat cultured cells obtained after 24 h of incubation. The treatment was done on a 96-well micro-titer plate and were further incubated for 72 h. A control, without addition of biosurfactant, i.e., only DMEM was also considered for better comparison. Following this, the medium was removed and MTT dye of 0.5 mg mL^−1^ final concentration was added in the wells and then incubated for 4 h. Finally, to dissolve the blue formazan precipitate, 100 mL of dimethylsulfoxide (DMSO) was added to each well and absorbance at 570 nm was measured by using a micro-plate reader (BioRad Model 680; Bio-Rad). The cell viability was expressed as a percentage of a control using the following equation,

Viability (%)=Nt/Nc×100

where, Nt denotes the absorbance of the cells treated with the biosurfactant, while Nc is the absorbance of the untreated control cells.

### Antimicrobial activity

The antimicrobial activity of the biosurfactant was estimated against four pathogenic strains of bacteria (*Escherichia coli, Staphylococcus aureus, Klebsiella pneumonia*, and *Bacillus subtilis*) and two pathogenic strains of fungi (*Aspergillus flavus* and *Aspergillus niger*) using agar well diffusion assay method given by Bharali et al. (2014) with slight modification (2014). Fifty mg L^−1^ of column purified biosurfactant was used for the study. Ciprofloxacin (10 μg/ disc) and Fluconazole (1.0 mg/disc) were used as a positive control for bacterial culture and fungal culture respectively. The antimicrobial activity of the biosurfactant was assessed by measuring the diameter of the zone of inhibition at cross angles.

### Statistical analysis

All of the experiments were carried out three times and studied in triplicate. Results represent the mean ± standard deviation. One-way analysis of variance (ANOVA) with the least significant difference (LSD) test was conducted to determine the significant differences in hydrocarbon degradation efficacy of the bacterial strain at different time periods. SPSS ver.18 software (Chicago, IL) was used to carry out the statistical analysis.

## Results and discussion

### Biosurfactant producing bacteria

Ten bacterial colonies of different morphology were isolated from the hydrocarbon contaminated soil sample followed by their screening for production of biosurfactant. Among the ten selected isolates, culture broth of five isolates showed positive results in the drop collapse assay, thereby indicating the presence of biosurfactant in the culture media. The drop of crude oil was collapsed immediately or within 1 min of addition of culture broth. The remaining bacterial culture broths could not collapse the drop of crude oil even after 1 min.

All five biosurfactant producer isolates that exhibit positive drop collapse assay were able to reduce the ST of the culture broth to <35 mN m^−1^ (Table 1). According to Joshi et al. (2008) isolates capable of reducing the ST of the medium to ≤ 35 mN m^−1^ can be considered to be strong biosurfactant-producing microbes (2008). Therefore, it was affirmed that five isolates namely, PG1, PG3, PG6, PG9, and PG10 are efficient biosurfactant producer.

**Table 1:** **ST (mN/m) of bacterial isolates on glucose-containing mineral medium at different time intervals**.

**Bacterial isolates**	**ST at**					
	**0 h**	**24th h**	**48th h**	**72th h**	**96th h**	**120th h**
C^a^	71.1 ± 0.30	71.0 ± 0.24	71.0 ± 0.40	69.9 ± 0.30	69.9 ± 0.23	69.8 ± 0.27
PG 1^*^	61.9 ± 0.40	46.4 ± 0.44	30.5 ± 0.43	33.0 ± 0.31	38.0 ± 0.33	43.2 ± 0.23
PG 2	59.1 ± 0.30	50.5 ± 0.25	46.5 ± 0.30	47.2 ± 0.25	49.7 ± 0.15	52.7 ± 0.25
PG 3^*^	60.8 ± 0.30	45.8 ± 0.44	28.4 ± 0.22	34.5 ± 0.23	36.9 ± 0.24	41.8 ± 0.28
PG 4	69.8 ± 0.12	68.5 ± 0.17	60.1 ± 0.24	60.0 ± 0.43	62.7 ± 0.41	62.8 ± 0.31
PG 5	60.8 ± 0.15	57.7 ± 0.21	54.4 ± 0.16	57.8 ± 0.23	57.0 ± 0.16	57.3 ± 0.26
PG 6^*^	62.9 ± 0.40	47.4 ± 0.21	33.4 ± 0.41	37.3 ± 0.12	41.2 ± 0.31	44.3 ± 0.22
PG 7	69.4 ± 0.16	59.2 ± 0.24	58.6 ± 0.22	57.1 ± 0.35	56.2 ± 0.40	56.1 ± 0.30
PG 8	68.8 ± 0.12	60.5 ± 0.17	54.1 ± 0.24	57.8 ± 0.43	60.7 ± 0.41	62.8 ± 0.31
PG 9^*^	59.4 ± 0.30	44.5 ± 0.25	32.5 ± 0.30	38.3 ± 0.25	42.8 ± 0.15	44.3 ± 0.25
PG 10^*^	60.9 ± 0.41	45.4 ± 0.23	34.3 ± 0.27	38.4 ± 0.32	42.4 ± 0.41	47.1 ± 0.12

*C^a^Abiotic control*.

* *Denotes the biosurfactant producing strains*.

*Results represented mean ± SD of five measurements*.

### Selection of the most efficient hydrocarbon degrading biosurfactant producer

**Figure 1:**
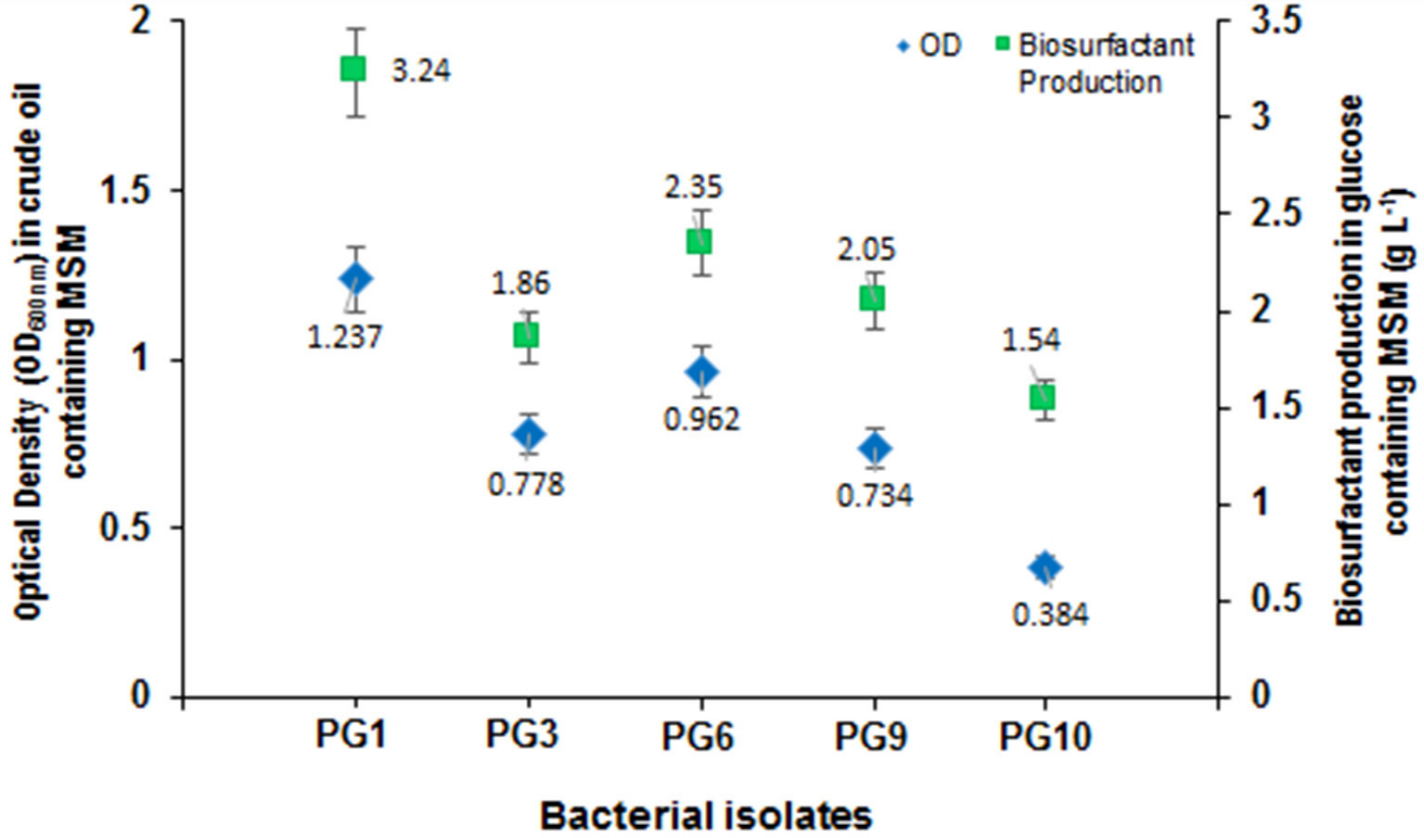
**Growth characterization of biosurfactant-producing isolates in mineral medium containing crude oil as the carbon source along with biosurfactant production (g/L) of each strain**. Bars represent the Standard error (SE) of three determinations.

From the total of five biosurfactant producing bacteria, isolate PG1 was selected as the most efficient crude oil degrader based upon its distinguished growth (OD_600nm_) on 2% (v/v) crude oil enriched condition (Figure 1). The isolate showed the maximum growth (OD_600_ = 0.824) in crude-oil containing media after 7th day of inoculation. Thus, from the results obtained, it was revealed that isolate PG1 achieved the best utilization of petroleum hydrocarbon through degradation. Again, isolate PG1could produce the highest amount of biosurfactant (3.24 g/l) on 2% (w/v) glucose containing MSM as compared to other biosurfactant-producing bacterial isolates grown in same culture conditions (Figure 1). Therefore, the isolate PG1 was selected for further degradation studies.

### Identification of the selected bacterial strain

Morphological and physiological characteristics of the isolate PG1 showed its resemblance with *P. aeruginosa* having smooth surfaced, small and raised colony morphology and gram negative rod shaped structure. The biochemical study also revealed the isolate to be a *P. aeruginosa* with positive catalase test, gelatinase test, oxidase test, hemolytic test, and negative glucose fermentation test. The 16S rRNA gene partial sequence of strain PG1 (966 nt) was submitted to the NCBI GenBank database under accession no. gb|**KU095843**|. BLAST search was conducted to compare the sequences with existing sequences and sequence similarity to the closest sequence of *P. aeruginosa* (accession no. gb|**CP008873**|) were found to be 99.90%. Therefore, the identity of the unknown strain was confirmed as *P. aeruginosa*PG1.

### Degradation of crude oil

**Figure 2:**
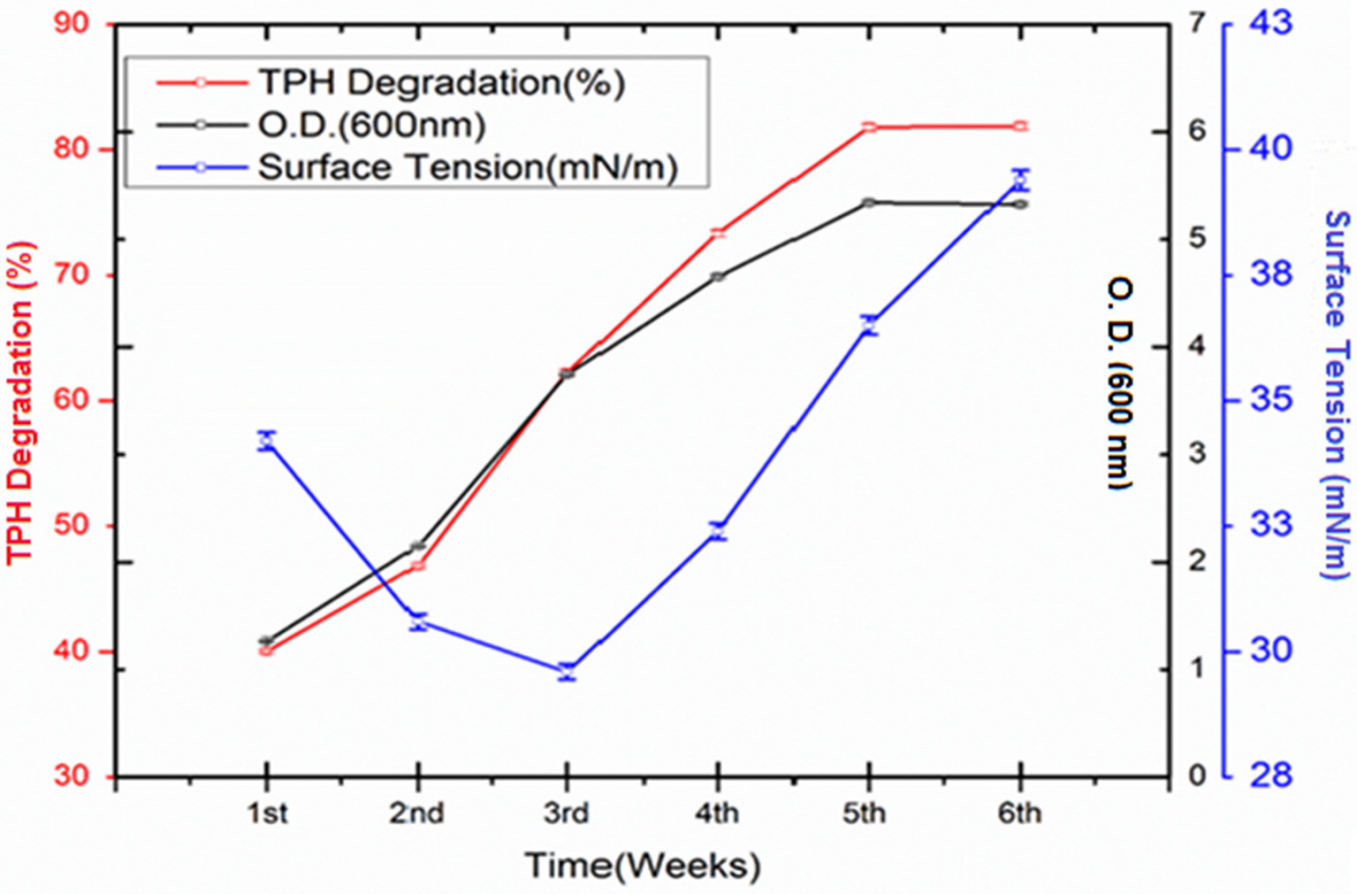
**Quantity of TPH degraded (%) by *Pseudomonas aeruginosa* PG1 at weekly intervals up to 6th week of incubation along with the growth of the consortium in the media**. Bars represent the ± standard deviation (± SD).

**Figure 3:**
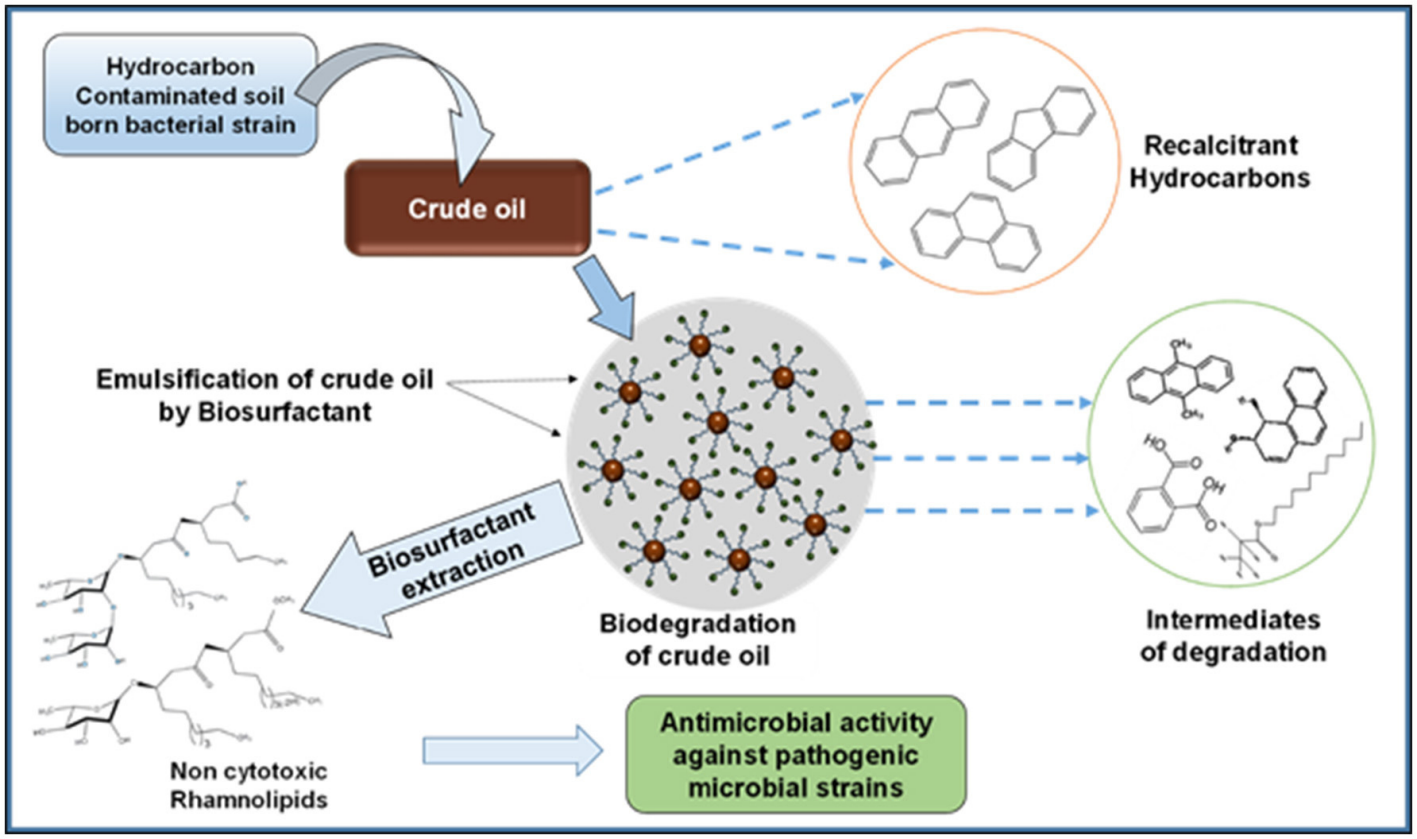
**Schematic presentation showing the activity of bacterial strain in degrading recalcitrant petroleum hydrocarbons with simultaneous production of biosurfactant**.

The crude oil degradation pattern of strain *P. aeruginosa* PG1 revealed that there was an increasing trend in degradation for every successive week up to the fifth week (81.8%) whereas minimum (40.36%) was recorded in the first week (Figure 2). The difference in TPH degradation values at different incubation period was statistically significant, however, there was no significant increment in degradation after fifth week of incubation (ANOVA LSD test, *p* < 0.05). It was also observed that during the degradation process of crude oil by the bacterial strain, the ST of the culture medium got reduced from 51.8 to 29.6 mN m^−1^ which signifies production of biosurfactant (Figure 2). The simultaneous production of biosurfactant in the culture broth with continuous crude oil degradation implies that the biosurfactant producing strain utilizes various crude oil components as substrates for the production of biosurfactant. This in turn boosts the overall degradation process of crude oil (Antoniou et al., 2015). It was observed that there was an increase in the values of ST after the 3rd week of culture (Figure 2). Most biosurfactants are considered to be secondary metabolites, however, some may be utilized for the survival of biosurfactant-producing microorganisms for facilitating nutrient transport (Rodrigues et al., 2006). The production of biosurfactant starts during stationary phase and continues up to death phase. The value of ST continues to decrease until the point of CMC (Critical micelle concentration), after which no further reduction occurs. The reason behind the increment in surface tension after the 3rd week of culture may perhaps be due to degradation of biosurfactant in the culture media or utilization of biosurfactant for microbial survival. The mode of the microbial action in degrading recalcitrant petroleum hydrocarbons and simultaneous production of biosurfactant in this course is presented here within a schematic diagram in Figure 3.

In this investigation, the concerned strain showed a significantly higher degradation of crude oil compared to many recent reports dealing with the microbial application for crude oil degradation (Sathishkumar et al., 2008). By employing two mechanisms biosurfactant enhances microbial degradation of hydrocarbon, namely, by increasing the bioavailability of substrate through emulsification and by facilitating association of hydrophobic substrates with bacterial cells through reduction of cell surface hydrophobicity of bacterial cell (Mulligan and Gibbs, 2004). Biosurfactant increases the surface areas of sparsely soluble hydrocarbon compounds by reducing surface and interfacial tensions which lead to increased bioavailability and mobility of contaminants (Mahanty et al., 2006). In consequence, biosurfactant enhances the rate of hydrocarbon bioremediation. Accordingly, by introducing biosurfactant producing bacteria to contaminated culture system, an enhanced biodegradation can be achieved through mobilization, solubilization, or emulsification of hydrocarbons (Nievas et al., 2008). In a crude oil degradation study by Kumari et al., it was reported that two biosurfactant producing strain, namely, *Pseudomonas* sp. BP10 and *Rhodococcus* sp. NJ2 degraded 60.6 and 49.5% of TPH respectively when incubated for 30 d at optimized culture conditions in MSM containing 2% of crude oil (Kumari et al., 2012). Tang et al. (2007) reported the enhanced crude oil degradation by strain *P. aeruginosa* ZJU which could produce rhamnolipid biosurfactant in glycerol containing culture medium, but the study haven't described anything about the biosurfactant production during crude oil degradation. Al-Wasify and Hamed (2014) described that their experimental bacteria *P. aeruginosa* could carry out a maximum degradation (77.8%) at 22°C after 28 days of incubation.

**Figure 4:**
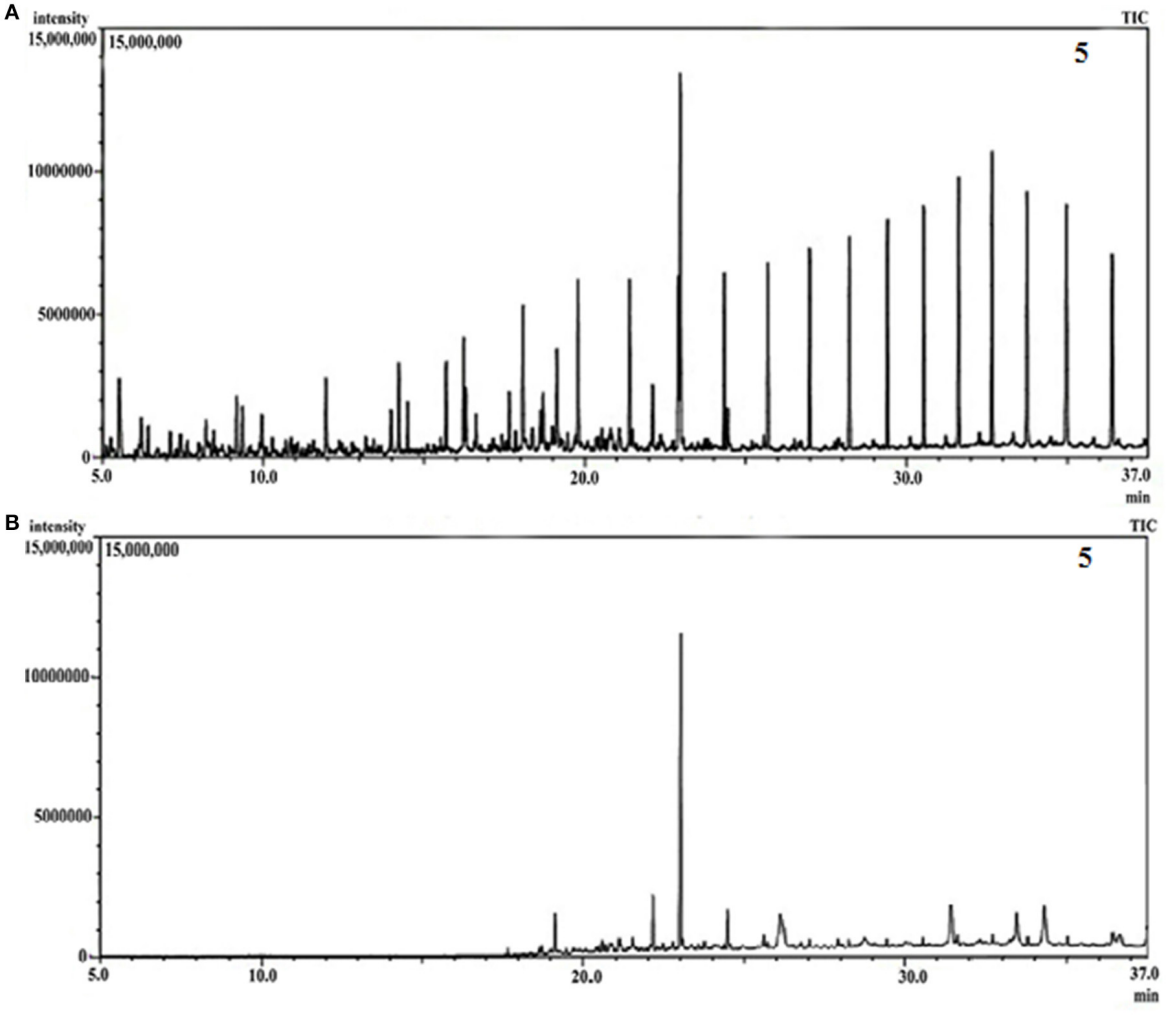
**(A)** GCMS chromatograph of abiotic control, **(B)** GCMS chromatograph of crude oil treated for 5 week with *Pseudomonas aeruginosa* PG1.

GCMS analyses of the residual hydrocarbon extracted from strain PG1 culture was conducted after 5 weeks and compared with an abiotic control assayed under the same conditions. The obtained chromatograms are presented in Figure 4. From the chromatograms, it was revealed that TPH is reduced in the sample treated with strain *P. aeruginosa* PG1 as compared to the abiotic control sample. It validates the gravimetric results and suggests that the strain was highly effective in degrading different components of crude oil. The microbial degradation of aliphatic hydrocarbon initiate with the terminal methyl group oxidation to form a primary alcohol which further gets oxidized to corresponding aldehyde, and finally to the fatty acid derivatives. Even so, in some instances, oxidation process involves both the end of alkane molecule and produces ω-hydroxy fatty acids. Through β-oxidation, these resultant ω-hydroxy fatty acids are further converted to dicarboxylic acids (Coon, 2005). Sub-terminal oxidation of n-alkanes generates secondary alcohols which convert to the corresponding ketone and then oxidized by Baeyer–Villigermonooxygenase to an ester. Enzyme esterase facilitates hydroxylation of the resulting ester producing an alcohol and a fatty acid [41]. Wide ranges of alkanes (C_8_ to C_36_) including both short chain and long chain alkanes namely, n-octane (C_8_), n-undecane (C_11_), 2 bromo- dodecan (C_12_), 2,6,10-Trimethyldodecane (C_15_), n-hexadecane (C_16_), pristane (C_19_), 3-methyl-nonadecane (C_20_), didecyleicosane (C_20_), heneicosane (C_21_), 2 methyl -tetracosane (C_24_), 3-methyl-octacosane (C_29_), n-nonacosane (C_29_), 3-methyl-nonacosane (C_30_), n-Tritriacontane (C_33_), and n-Hexatriacontane (C_36_) that were present in the abiotic control sample were degraded by the bacterial strain. Along with other aromatic hydrocarbons, six different PAHs were detected in the untreated crude oil. From the six different PAHs detected in untreated crude oil, the bacteria were able to completely degrade two of them, namely, 1H-Indene and 3-beta-Myristoylolean-12-en-28-ol. The other four PAHs, Naphthalene, Fluorene, Phenanthrene, and Anthracene along with their various derivatives were also reduced to significantly lower concentration, but not completely degraded after the treatment with strain PG1 (Table 2). Pasumarthi et al. (2013) investigated the crude oil degradation ability of a newly isolated enrichment culture containing two bacterial strains of *P. aeruginosa* and *Escherichia fergusonii*. The study was carried out in naturally contaminated soil microcosm and it was revealed that the enrichment culture was able to degrade n-alkanes (ranging from C_12_ to C_33_) at a higher rate as compared to PAHs. However, PAHs like napthalene, fluorine, anthracene, and their various derivatives were moderately degraded by the bacterial culture.

**Table 2:** **Comparison of the PAHs detected in crude oil sample treated with strain PG1 for 5 weeks and the untreated abiotic control crude oil sample**.

**Name of PAHs**	**Abiotic control crude oil**	**Crude oil treated for 5 week by strain PG1**	**Degradation percentage (%)**
Naphthalene	Present (7.46)	Present (1.89)	74.67
Fluorene	Present (1.84)	Present (0.49)	73.37
Phenanthrene	Present (1.78)	Present (0.52)	70.79
Anthracene	Present (1.96)	Present (0.62)	68.37
3- 3-beta-Myristoylolean-12-en-28-ol	Present (0.37)	Absent	100
1H-Indene	Present (0.28)	Absent	100

*Values in parenthesis denotes the total area % of respective hydrocarbons*.

In the treated sample some new compounds were observed showing the generation of 12 prominent degradation intermediates forming various esters and acids. Different degradation intermediates were detected in the treated sample are (a) Cyclohexylmethyl oxalic acid; (b) 10-chlorodecyl formic acid ester; (c) Carbonic acid, 2-biphenyl ester; (d) Octanoic acid 2-pentadecyl ester; (e) 9-10-Dimethylanthracene; (f) Hexa-decanoic acid, methyl ester; (g) octacosanoic acid methyl ester; (h) 3,4-dihydroxy-phenanthrene diol; (i) 2,4,5 trifluorobenzyl alcohol; (j) Propanedioicacid, dipropyl, dimethyl ester; (k) Phthalic acid ester; and (l) Oxalic acid, cyclohexyl-methyl-tridecyl ester. The emergence of these new peaks might have resulted from two phenomena, either from the microbial degradation of hydrocarbons or the synthesis of intermediates and new metabolites during the fermentation process (Seo et al., 2009; Singh et al., 2012; Patowary et al., 2016b).

### Extraction of crude biosurfactant and CMC determination

The amount of biosurfactant produced in the course of crude oil degradation was 2.26 g L^−1^. The biosurfactant that produced during the degradation process had the ability to reduce the surface tension from 51.8 to 29.6 mN m^−1^ of the growth medium. The color of the crude biosurfactant was honey brown.

The CMC value of the extracted biosurfactant was also calculated (Supplementary Figure S1). The surface tension reduction ability of a surfactant largely depends on the CMC. At CMC, surfactant molecules aggregate and form micelles in polar or aqueous environment. It was observed that the surface tension speedily diminishes as the concentration of the biosurfactant was increased and a minimum surface tension reading of 28.6 mN m^−1^ was obtained at 56 mg L^−1^ concentration. There was no further decrease in surface tension even after increasing the concentration of the biosurfactant beyond 56 mg L^−1^. Therefore, the CMC value of the crude biosurfactant was calculated to be ~56 mg L^−1^ from the break point of surface tension vs. its log of concentration curve. The result indicated that the crude biosurfactant possesses exquisite surface tension property along with a lower value of the CMC.

### Characterization of the biosurfactant

#### Biochemical

In the ninhydrin test, formation of Ruhemann's purple complex was absent implying the absence of protein or amino acid in the extracted biosurfactant. A bluish green color formation was noted in the anthrone test, which denotes the existence of carbohydrates in the biosurfactant. NaOH saponifies the lipid portion existing in the biosurfactant implying the presence of lipids in the biosurfactant. The rhamnose test which was performed for quantification of rhamnolipid in the biosurfactant samples, shows that 1 g L^−1^ of the crude biosurfactant produced by *P aeruginosa* PG1 was equivalent to 0.67 g L^−1^ of rhamnolipid. Thus, from the above biochemical assays, it can be concluded that the biosurfactants produced by the strain is rhamnolipid in nature.

#### FTIR

The FTIR spectrum of column purified biosurfactant revealed important bands at 3376, 2928, 1732, 1648, and 1038 cm^−1^ (Supplementary Figure S2). For interpretation of various functional groups present in the biosurfactant, the FTIR spectrum was compared with Pornsunthorntawee et al. (2008). Due to the presence of hydrogen bonding, the appearance of a strong and broad band of the hydroxyl group (-OH) free stretch was observed at 3376 cm^−1^. The occurrence of C-H stretching vibrations of hydrocarbon chain of alkyl (CH_2_-CH_3_) groups was confirmed by the absorption band observed at 2928 cm^−1^. Characteristic carbonyl stretching band which denotes the presence of ester compounds was found at 1732 cm^−1^. The stretching of COO^−^ group was asserted through the deformation vibration at 1648 cm^−1^.The absorption band found at 1038 cm^−1^ is the characteristics of the glycosidic bond (C-O-C) present in the molecule. Therefore, from the above discussion it can be summarized that the chemical structure of this biosurfactant is identical to those of previously reported rhamnolipid which comprises of rhamnose ring attached with long hydrocarbon chains.

#### LC-MS

**Figure 5:**
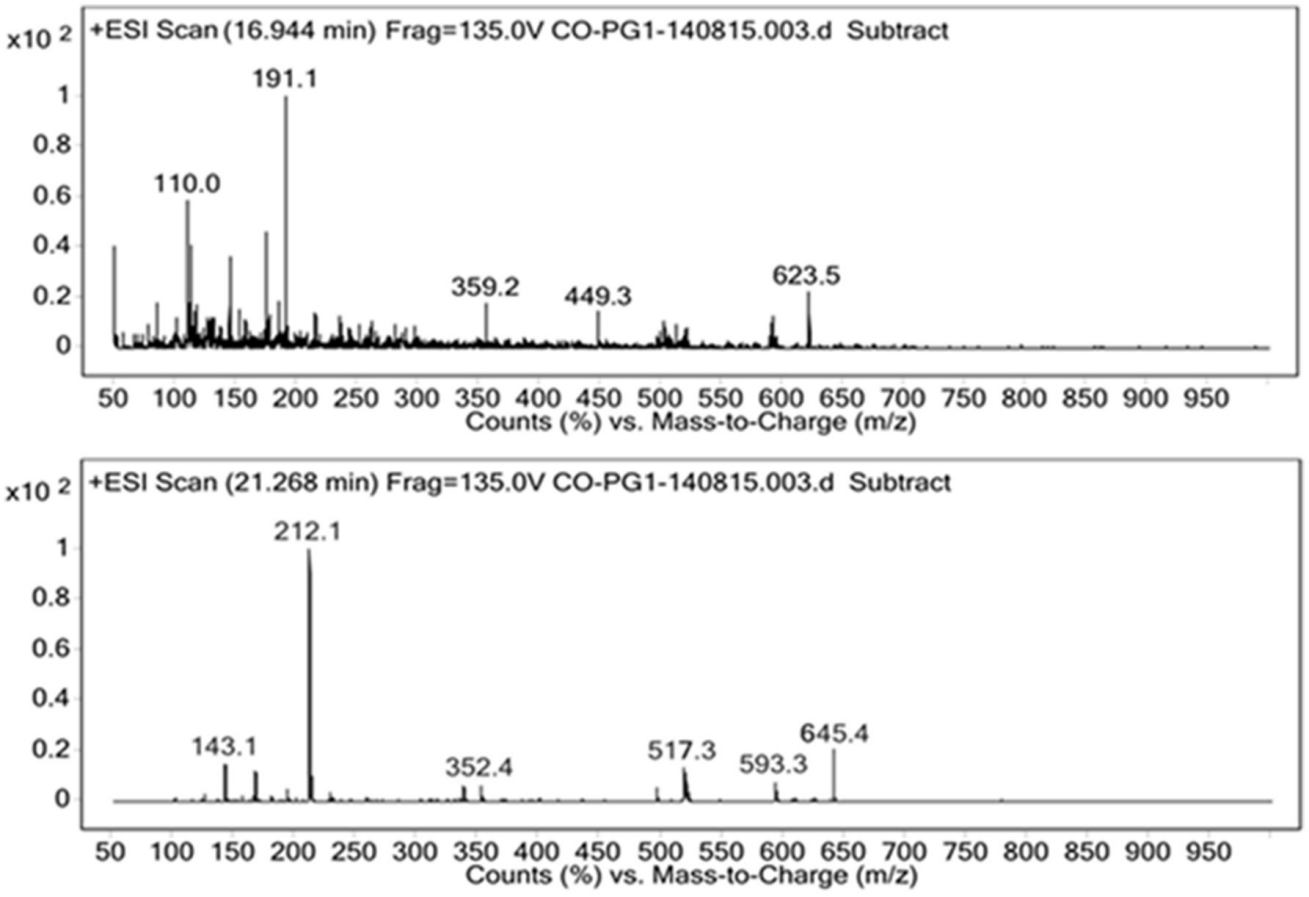
**Positive ion mode ESI-MS from the biosurfactant produced by *P. aeruginosa* PG1 grown in crude oil containing MSM**.

**Figure 6:**
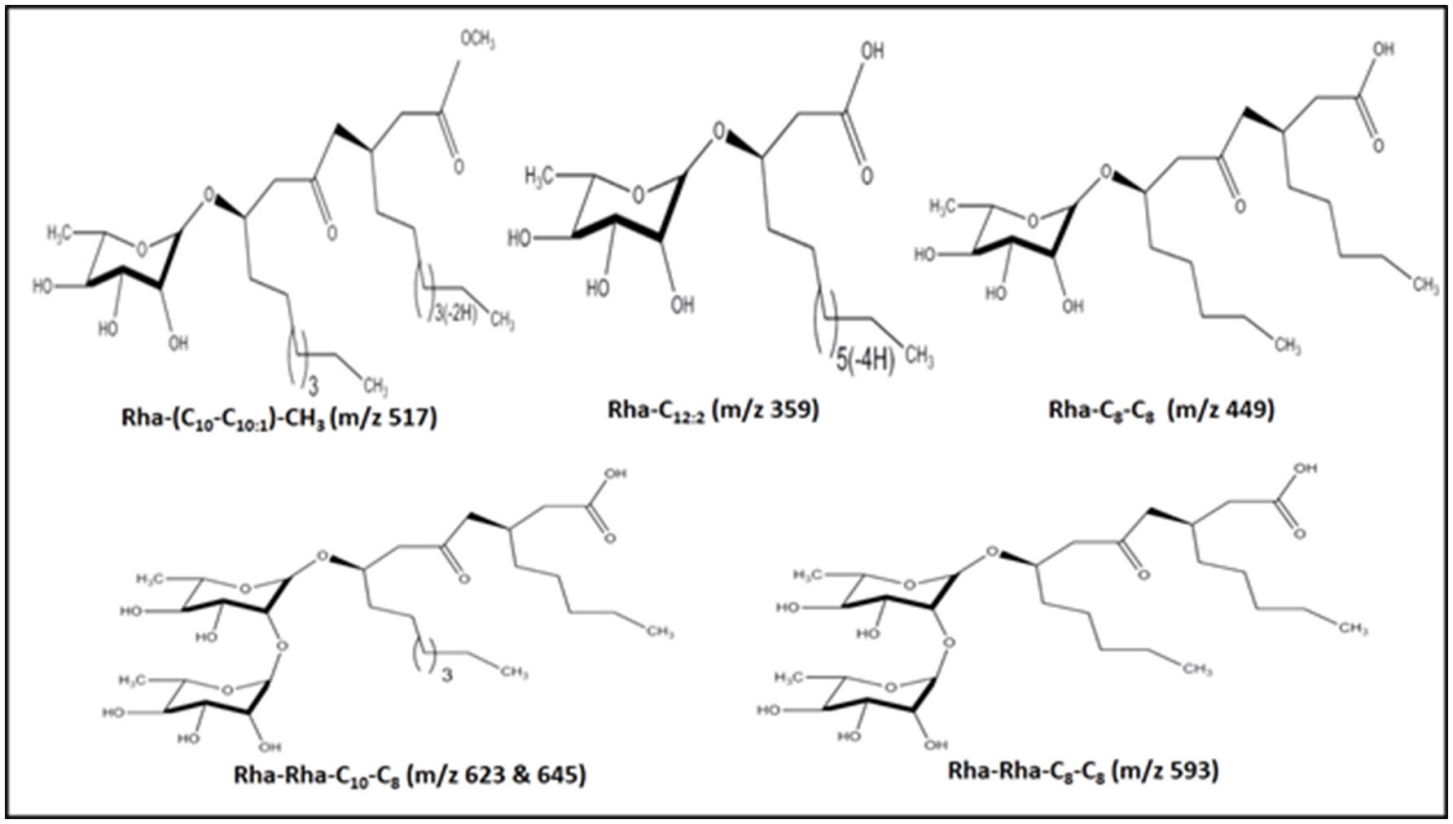
**Structures of the five rhamnolipid congeners detected in the column purified extract of rhamnolipid through LC-MS**.

For the identification of the structural constituents of the column purified biosurfactant, LC-MS analysis of the same was performed in positive ion mode. On comparison of the LC-MS data obtained for the biosurfactant, with those reported in previous literature, five rhamnolipid congeners were detected in the column purified biosurfactant (Figure 5) (Abdel-Mawgoud et al., 2010; Pantazaki et al., 2011). A methylated mono- rhamnolipid congener of m/z 517 was detected in the spectrum corresponding to [Rha-(C_10_-C_10:1_)-CH_3_]. Prominent peaks at m/z 359 and 449 were also observed which corresponds to [M+H]^+^ ion of [Rha-C_12:2_] and [Rha-C_8_-C_8_] respectively. The characteristic ions in MS at m/z 623 and 645 correlates to the molecular [M+H]^+^ ion and a sodium adduct [M+Na]^+^ ion of the same dirhamnolipid congener [Rha-Rha-C_10_-C_8_].Another di-rhamnolipid congener with m/z 593 was present in the spectrum which correlates to the deprotonated molecule [M-H]^−^ of [Rha-Rha-C_8_-C_8_]. Hence, it can be concluded that the biosurfactant produced by *P. aeruginosa* PG1 strain, utilizing crude oil as sole carbon source was an amalgamation of both mono and di-rhamnolipid (Figure 6). According to the available literature, various strains of *P. aeruginosa*are recognized to produce a mixture of different mono and di-rhamnolipid congeners in natural conditions (Patowary et al., 2016a). As stated by literature, various mono-rhamnolipid and di-rhamnolipid congeners of rhamnolipid biosurfactants produced by *P. aeruginosa* are essentially responsible for enhanced biodegradation of hydrocarbon pollutants like hexadecane, phenanthrene, pyrene and various other components of crude oil as a result of their physicochemical and microbiological effects on the availability of the contaminants (Hwang and Cutright, 2002; Noordman et al., 2002; Das et al., 2014).

#### SEM-EDS

The SEM-EDS analysis of the purified biosurfactant sample reveals the existence of carbon, oxygen, sodium, phosphorus, chlorine, and potassium in a ratio of 67.14, 30.73, 1.02, 0.29, 0.62, and 0.19 % in the scanned area (Supplementary Figure S3). The presence of carbon and oxygen in a comparatively profuse amount indicates the existence of carbohydrate and lipid complex moiety in the biosurfactant sample.

### Emusification activity

**Figure 7:**
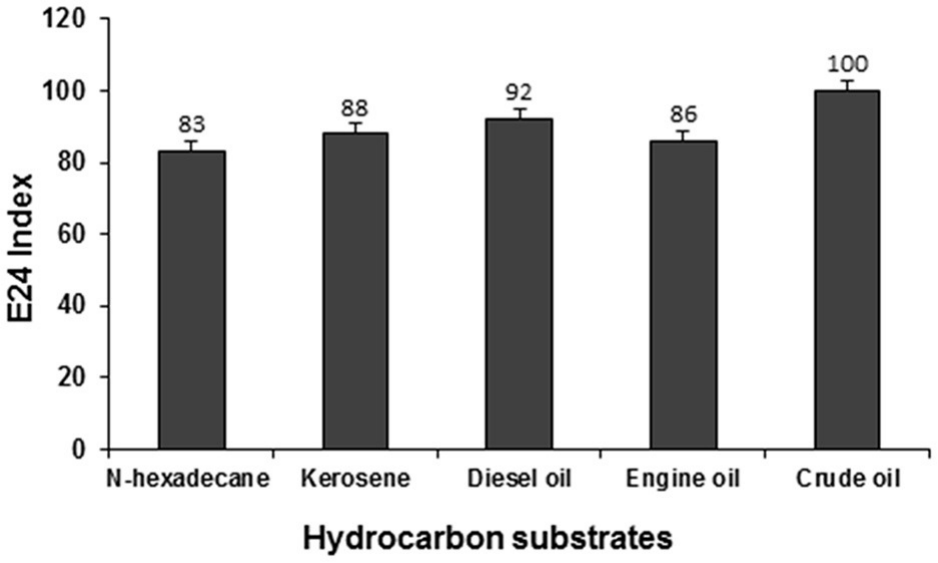
**Emulsification activity of the biosurfactant containing supernatant of PG1 against different hydrocarbon substrates**.

The emulsification activity of the supernatant culture of strain PG1 grown in crude oil containing medium was evaluated against n-hexadecane, kerosene, diesel oil, engine oil, and crude oil and their E24 indices were measured to be 83, 88, 92, 86, and 100% respectively (Figure 7). The maximum emulsification activity of the biosurfactant was 100% for crude oil, followed by other hydrocarbons in the order of diesel, kerosene, engine oil, and n-hexadecane. The biosurfactant sample demonstrated maximum emulsification activity against crude oil. Strong emulsification activity of a hydrocarbon degrading bacteria is always considered to be a very imperative aspect in the context of hydrocarbon degradation (Banat et al., 2000).

### Cytotoxicity study of the biosurfactant

**Figure 8:**
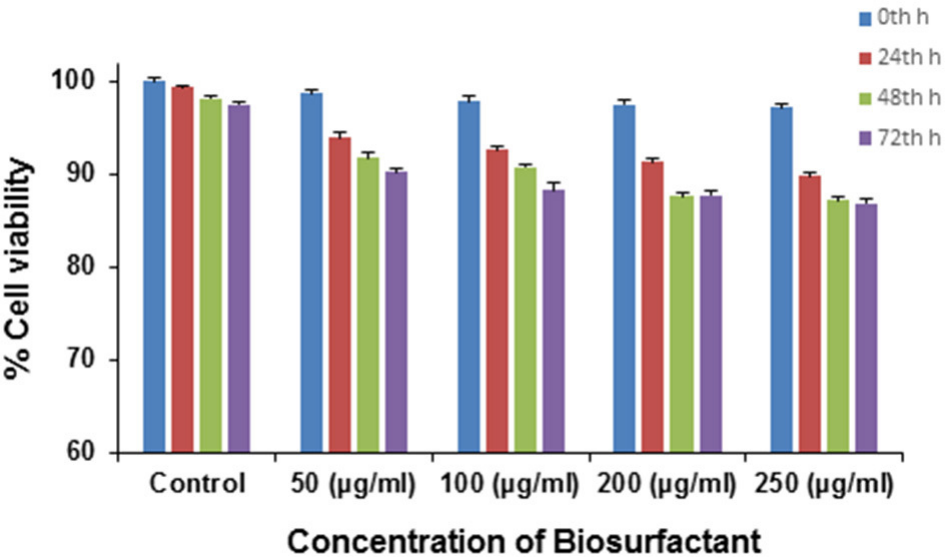
**Cytotoxicity of different doses of biosurfactant upon treating the L292 cell line in terms of the percentage of cell viability**.

Various microbial metabolites often possess an adverse effect on host organisms triggering many epidemic diseases including cytotoxic and neurotoxic effects. By and large various strains of *P. aeruginosa* are usually known for its production of toxic substances like exotoxins. However, in the present case, the application of the biosurfactant produced by *P. aeruginosa*PG1 strain utilizing crude oil as sole carbon source showed no cytotoxic effect on the mouse fibroblast L292 cell line which signified it to be a bench-mark as non-cytotoxic rhamnolipid that could be used as a possible biological material, for uses including biological interfaces. At 0th h of the treatment, the cell viability was 96.4% in case of 250 μg/ml (maximum concentration), whereas 100 % cell viability was achieved in case of the control. As the time of incubation increases, the cell viability reduces and after 72 h of incubation it was observed that viability of cells in the control reduces to 97.5%, whereas in case of 250 μg/ml (maximum concentration), the viability reduced to 85.6% (Figure 8). In pursuant to ISO 10993-5, 2009, cell viability above 80% can be considered as non-toxic in nature (ISO Report, 2009). Through an MTT dye conversion assay it was observed that the mouse fibroblastic cell-line were viable, with a viability range of 93.2 to 85.4 % on being treated with 50 to 250 μg of the purified biosurfactant in a micro-titer plate (Figure 8). This confirms the possible utility of this biosurfactant which acquire the safety standards for living organism.

### Antimicrobial activity

The biosurfactant exhibited antimicrobial activity toward the tested pathogenic strains of both bacterial and fungal culture (Table 3). The susceptibility of *B. subtilis, S. aureus, K. pneumonia*, and *E. coli* showed that the biosurfactant contained antibacterial properties that inhibited both Gram +ve and Gram –ve strains. Furthermore, it also inhibited the growth of both the pathogenic fungus. As seen in Table 3, the biosurfactant possesses significant antimicrobial activity against all the tested strains. These antibiosis results are the agreement with existing reports of antibiotic effect exhibited by rhamnolipid biosurfactant (Vatsa et al., 2010).

**Table 3:** **Antimicrobial activity of the purified biosurfactant**.

**Test organisms**	**Biosurfactant**	**Positive control**	**Negative control**
*B. subtilis*	9.64 ± 0.52	11.95 ± 1.62	0
*S. aureus*	7.42 ± 1.29	13.21 ± 1.28	0
*K. pneumoniae*	8.74 ± 0.35	14.64 ± 1.32	0
*E. coli*	6.73 ± 0.72	15.48 ± 1.42	0
*A. flavus*	7.32 ± 2.52	13.72 ± 1.86	0
*A. niger*	6.85 ± 2.18	13.62 ± 2.26	0

*Values are expressed as mean ± SD (n = 3)*.

*Zone of inhibition not include the diameter of the well (7 mm)*.

## Conclusion

The present study reported the biodegradation of crude oil by *P. aeruginosa* PG1isolated from hydrocarbon contaminated garage soil. Strain PG1 was found to be an efficient crude oil degrader and could produce rhamnolipid biosurfactant using crude oil as the sole carbon and energy source in the course of degradation. The strain exhibited excellent degradation of various crude oil components including a number of recalcitrant PAHs. The biosurfactant possesses high surface activity and exhibited excellent emulsification activities against different hydrocarbon substrates. The biosurfactant demonstrated negligible cytotoxic effect on testing with the L292 cell line, however significant antibiotic activity was obtained against some pathogenic strain of gram positive and gram negative bacteria and fungi. All these favorable properties facilitate the strain as an efficient tool in various environmental applications, particularly in the remediation of crude oil contamination sites.

## Author contributions

KP performed all the experiments, coordinated the data analysis, and prepared the manuscript. RP contributed in the preparation of the manuscript and data analysis. MK provided the research work suggestion. SD designed the research plan and supervised the whole study.

## Funding

The study was financially supported by the core fund of Institute of Advanced Study in Science and Technology (IASST), an autonomous institute under DST (Department of Science & Technology, Govt. of India).

### Conflict of interest statement

The authors declare that the research was conducted in the absence of any commercial or financial relationships that could be construed as a potential conflict of interest.
