# Supplementary material for: Characterization of Biosurfactant Produced during Degradation of Hydrocarbons Using Crude Oil As Sole Source of Carbon
Source: Front Microbiol. 2017 Feb 22;8:279. doi: 10.3389/fmicb.2017.00279 (PMC5319985; doi:10.3389/fmicb.2017.00279)
Supplement: Supplementary file 1 [file DataSheet1.PDF]

Supplementary Figure captions

Figure S1 Critical micelle concentration (CMC) of extracted biosurfactant produced by *P. aeruginosa* PG1.

Figure S2 Fourier transform infrared spectroscopy spectra of the crude biosurfactant obtained from strain PG1.

Figure S3 (a) Image of purified biosurfactant using EDS and (b) EDS spectra of the biosurfactant indicating the presence of C, O, Na, Si, Cl and K in the scanned area.

Figure S1

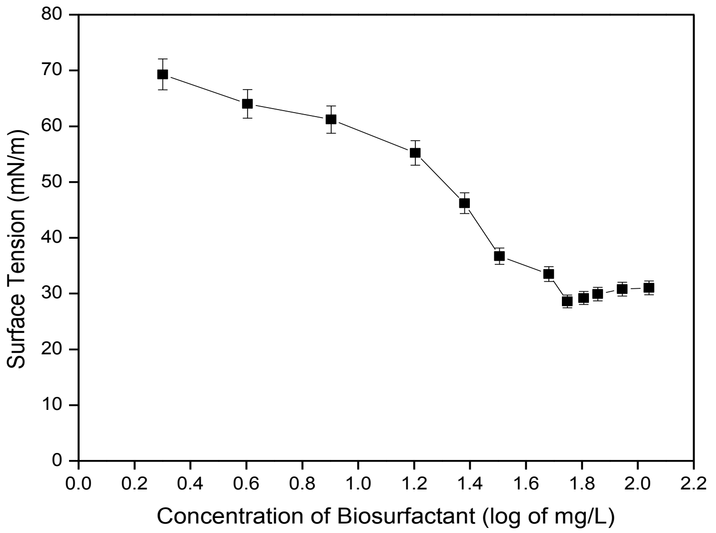

Surface Tension (mN/m)
0
10
20
30
40
50
60
70
80
0.0
0.2
0.4
0.6
0.8
1.0
1.2
1.4
1.6
1.8
2.0
2.2
Concentration of Biosurfactant (log of mg/L)

Figure S2

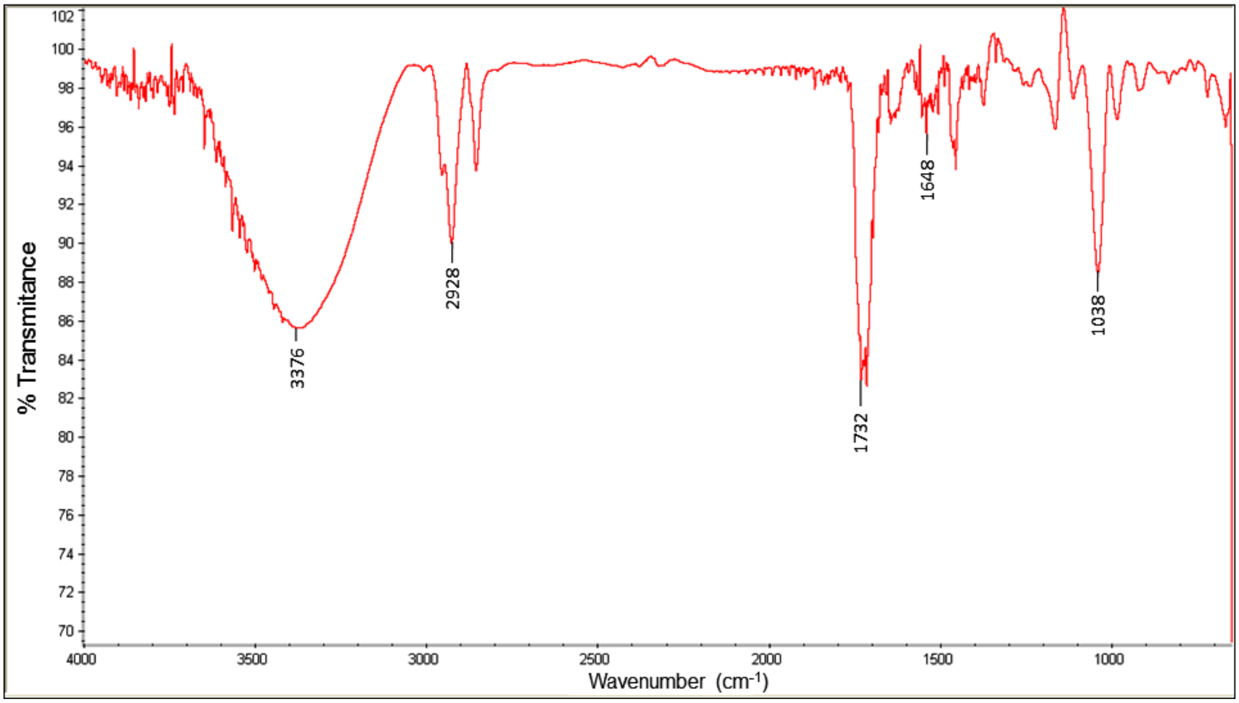
% Transmittance
Wavenumber (cm-1)
3376
2928
1732
1648
1038
4000
3500
3000
2500
2000
1500
1000
102
100
98
96
94
92
90
88
86
84
82
80
78
76
74
72
70

Figure S3

(a)

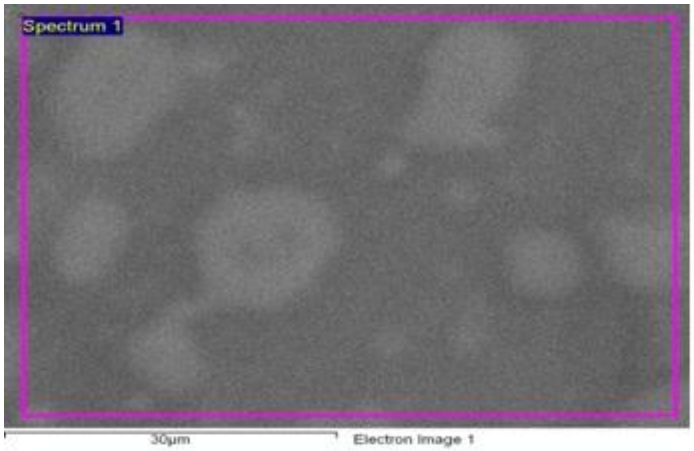

Spectrum 1
30µm
Electron Image 1

(b)

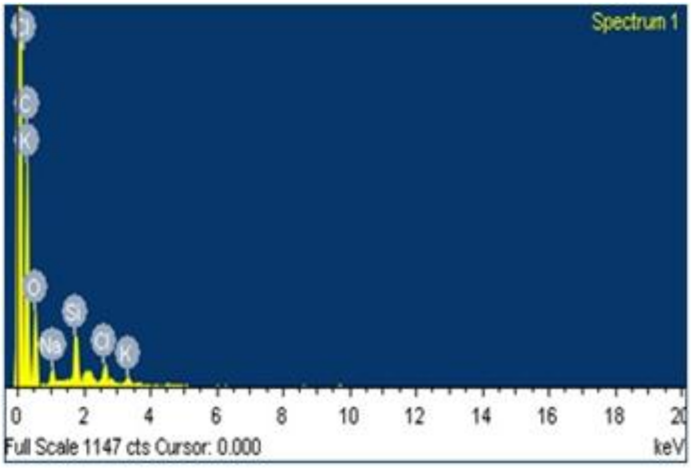

Spectrum 1
Cl
C
K
O
Si
Na
Cl
K
0
2
4
6
8
10
12
14
16
18
20
Full Scale 1147 cts Cursor: 0.000
keV
